# Positron emission tomography/computed tomography outperforms MRI in the diagnosis of local recurrence and residue of nasopharyngeal carcinoma: An update evidence from 44 studies

**DOI:** 10.1002/cam4.1882

**Published:** 2018-12-21

**Authors:** Zhanzhan Li, Yanyan Li, Na Li, Liangfang Shen

**Affiliations:** ^1^ Department of Oncology, Xiangya Hospital Central South University Changsha Hunan Province China; ^2^ Xiangya Hospital Central South University Changsha Hunan Province China

**Keywords:** magnetic resonance imaging, nasopharyngeal carcinoma, positron emission tomography/computed tomography, recurrence/residue, sensitivity, specificity

## Abstract

Studies on nasopharyngeal carcinoma (NPC) in five electronic databases were systematically searched online from the inception to June 5, 2018. Quality of the included studies was assessed using the updated Quality Assessment of Diagnostic Accuracy Studies 2. Data of sensitivity, specificity, positive likelihood ratio, negative likelihood ratio, diagnostic odds ratio, and the 95% confidence intervals were pooled using a bivariate random‐effect model. Forty‐four studies with 61 groups of data and totally 3369 patients were included in the qualitative and quantitative synthesis analysis. The overall estimated sensitivity and specificity of positron emission tomography/computed tomography/magnetic resonance imaging (PET‐CT/MRI) for local recurrent/residual NPC were 0.90 and 0.85, respectively. The pooled area under the curve of (AUC) of PET‐CT/MRI in the summary receiver operator characteristic curve was 0.94. Subgroup analysis showed MRI vs PET‐CT had lower sensitivity (0.83 vs 0.92) and specificity (0.78 vs 0.89). The AUCs of MRI and PET‐CT were 0.87 and 0.96, respectively. No‐cross of 95% CI was found in MRI vs PET/CT (0.87‐0.90 vs 0.94‐0.98). Meta‐regression showed PET/CT vs MRI was a potential source of heterogeneity. PET/CT and MRI both showed quite high overall ability in diagnosing local recurrent/residual NPC, but the subgroup analysis indicated PET‐CT was superior over MRI in diagnosis of local recurrence and residue of NPC after radiotherapy. The examination methods affected the heterogeneity within studies.

## 1 INTRODUCTION

Nasopharyngeal carcinoma (NPC) is a common malignancy in the head and neck with significant regional and ethnic distribution. South China and Southeast Asia are among the pandemic areas of NPC.^1^ However, pathogenesis of NPC has not been fully understood. Etiology and pathogenesis studies suggest the causal factors of NPC may include Epstein‐Barr virus infection, genetic factors, chemical carcinogens, and disturbance in oncogenes and tumor suppressor genes.^2^, ^3^, ^4^, ^5^ Due to the specific anatomical structure and position, NPC is preferentially treated by radiotherapy,^6^, ^7^ which has greatly improved the remission rate of NPC treatment and raised the overall average of 5‐year survival rate to over 70%.^8^ However, residues, local recurrence, and metastasis still impede the prognosis of NPC patients and limit further improvement in survival. Thus, it is of great importance to accurately and early identify the residues and recurrence of NPC.

However, some side effects would appear after radiotherapy, such as edema, inflammation, fibrosis, and scar.^9^ The resulting morphological changes could make traditional examination methods such as computed tomography (CT) and magnetic resonance imaging (MRI) insensitive to recurrence and residues and cause false positive or negative diagnosis.^10^ In recent years, 18F‐fluorodeoxyglucose (or 18‐fludeoxyglucose; 18F‐FDG) positron emission tomography (PET)/CT has been implemented. The perfect combination of CT morphological imaging and PET functional metabolic imaging has increased the sensitivity and specificity to lesions. Moreover, the overall diagnostic value of MRI and PET/CT in diagnosing local residual and recurrent NPC has been summarized, but this review only includes 14 studies.^11^ In the current study, we systematically searched several online databases and included 44 studies involving 61 groups of data in order to more accurately estimate the diagnostic ability of PET/CT and MRI for local recurrent and residual NPC.

## 2 METHODS

This study follows the Cochrane Handbook for Systematic Reviews and the Preferred Reporting Items for Systematic Reviews and Meta‐analysis (PRISMA, Data S1).^12^ No ethical approval was applicable for this secondary study based on previous articles.

### 2.1 Search strategy

PubMed, Web of Science, EMBASE, China National Knowledge Infrastructure, and Wanfang were systematically searched online from the inception to June 5, 2018. The following medical subject heading terms and keywords were used: (“nasopharyngeal carcinoma” OR “nasopharynx cancer OR “NPC”) AND (“positron emission tomography” OR “PET” OR “PET/CT” OR “PET‐CT” OR “18‐fluoro‐2‐deoxyglucose positron emission tomography” OR “18F‐FDG PET/CT” OR “MRI” OR “magnetic resonance imaging” OR “nuclear magnetic resonance scanner” OR “magnetic resonance angiography”) in combination with some keywords: recurrent or recurrence, residue, diagnosis or diagnostic (sensitivity and specificity), receiver operating curve or ROC. The references of some reviews and articles were also reviewed in order to obtain the potentially eligible trials. Languages were restricted to Chinese and English.

### 2.2 Study selection

Two authors independently scanned and screened the titles, abstracts, and full texts of the initially retrieved studies. Disagreements were resolved by explicit consensus. The inclusion criteria were as follows: (a) Recurrence and residues of NPC were confirmed by golden standard (biopsy or follow‐up); (b) the aim was to assess the diagnostic ability of PET/CT or MRI or both for recurrence or residues or both of NPC; (c) enough data were provided for further pooling analysis, including true positive (TP), false positive (FP), false negative (FN), and true negative (TN). For duplicates, the latest publication was used. Studies with duplicated or unqualified data, or focused on animal or experimental design were excluded. Reviews, comments, letter, and case reports were also excluded.

### 2.3 Data extraction

Two authors independently extracted data and resolved discrepancies by mutual discussion. From each included study, the following information was extracted: surname of first author, year of publication, country, examination method (PET/CT vs MRI), study design (prospective vs retrospective), age (range, mean or median), time of examination, golden standard (biopsy vs follow‐up), sample size, four folds data (TP, FP, FN, TN), sensitivity, and specificity. The extracted data were put into a standardized Excel sheet.

### 2.4 Assessment of quality

Quality of the included studies was assessed using the updated Quality Assessment of Diagnostic Accuracy Studies 2, which consists of two parts: risk of bias and applicability concerns. The risk of bias includes four items: patient selection, index test, reference standard flow, and timing. Each item has three options: high, unclear, and low. A study with ≥1 item scored “high” is considered as high risk of bias, whereas a study with all items scored “low” is treated as low or unclear risk of bias. The applicability concerns consist of three options: high, low, and unclear.^13^

### 2.5 Statistical analysis

Statistical analyses were performed on Stata 13 (StataCorp LP, College Station, TX, USA), and the quality was assessed on Review Manager 5 (Nordic Cochrane Centre, Cochrane Collaboration, 2014). Firstly, the threshold effect was evaluated by Spearman correlation coefficient, significant value of which means the existence of the threshold effect.^14^, ^15^, ^16^ Heterogeneity was evaluated by chi‐square and *I* ^2^ statistic, with the significance level at *P* < 0.05 or *I* ^2^ > 50%.^17^ Sensitivity, specificity, positive likelihood ratios (PLR), negative likelihood ratio (NLR), diagnostic odds ratio (DOR), and the 95% confidence intervals (CIs) were pooled using a bivariate random‐effect model.^18^ The summary receiver operator characteristic curves (SROCs) were also plotted. Subgroup analyses were conducted by population (China vs other countries), sample size (≤45 vs >45), examination methods (MRI vs PET/CT), study design (prospective vs retrospective), and golden standard (biopsy, follow‐up, or both). The potential influencing factors of heterogeneity were explored through meta‐regression involving the variables of publication year, country, examination methods, study design, golden standard, and sample size. Publication bias was evaluated via Deek's linear regression^19^ with the significance level at *P* < 0.05.

## 3 RESULTS

### 3.1 Study selection

**Figure 1:**
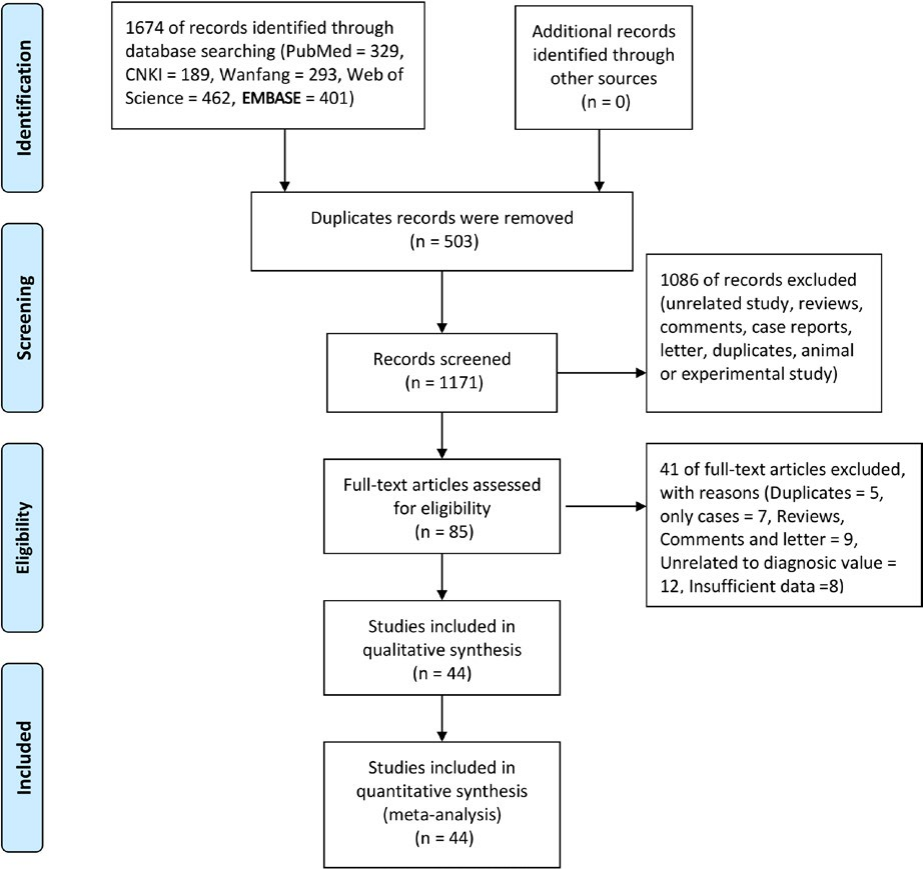
Flow diagram of studies’ selection process

Figure 1 presented the flow of study selection. The initial search returned 1674 records (PubMed = 329, CNKI = 189, Wanfang = 239, Web of Science = 462, EMBASE = 401). After 503 duplicates were removed, the remaining 1171 records were screened by scanning titles and abstracts, which excluded 1086 records because of unrelated topic, reviews, comments, case reports, or animal or experimental study. Then, 85 full‐text articles were left for eligibility assessment, which excluded 41 studies, including five duplicates, seven cases, nine reviews, comments or letters, 12 studies unrelated to diagnostic value, and eight studies with insufficient data. Finally, 44 studies involving 61 groups of data and 3369 patients were included in the qualitative and quantitative synthesis analysis (Data S2).

### 3.2 General characteristics of included studies

The general characteristics of the included studies were presented in Table 1. These studies were published from 1991 to 2016. The sample sizes ranged from 17 to 179 patients. The 61 groups of data were from China (55), Turkey (2), Singapore (2), Italy (1), and Saudi Arabia (1), adopted MRI (23) and PET/CT (38), and were prospective (29) and retrospective (32). The golden standard of the 61 groups of data was biopsy (10), follow‐up (8), or both (43). The median sample size was 45, but was <45 and >45 in 31 and 30 groups of data, respectively. The sensitivity and specificity of the included studies ranged from 55.6% to 100.0% and from 15.4% to 100.0%, respectively.

**Table 1:** Characteristics of the included studies

Author	Year	Country	Examination	Study design	Age (years)	Time (month)	Golden standard	Sample size	TP	FP	FN	TN	Sensitivity, %	Specificity, %
Gong	1991	China	MRI	Prospective	14‐62	—	Follow‐up	66	26	5	0	35	100.0	87.5
Kostakoglu1	1997	Turkey	MRI	Prospective	15‐76	3	Biopsy	17	3	5	0	9	100.0	64.3
Kostakoglu2	1997	Turkey	MRI	Prospective	15‐76	6	Biopsy	18	5	11	0	2	100.0	15.4
Chong1	1997	Singapore	MRI	Prospective	28‐67	>5.1	Biopsy	45	5	6	4	30	55.6	83.3
Chong2	1997	Singapore	MRI	Prospective	28‐67	>5.1	Biopsy	45	5	8	4	28	55.6	77.8
Peng	2000	China	PET	Retrospective	33‐62	≥6	Biopsy or follow‐up	32	11	0	1	20	91.7	100.0
Jiang	2000	China	MRI	Retrospective	19‐62	≥4	Biopsy or follow‐up	77	21	7	13	36	61.8	83.7
Chen	2002	China	PET	Retrospective	31‐65	≥2	Biopsy or follow‐up	25	14	4	2	5	87.5	55.6
Tsai	2002	China	PET	Prospective	19‐75	≥4	Biopsy or follow‐up	28	13	1	0	14	100.0	93.3
Kao	2002	China	PET	Prospective	18.8‐67	≥4	Biopsy	36	11	1	0	24	100.0	96.0
Wu1	2003	China	PET	Retrospective	23‐75	≥10	Biopsy or follow‐up	32	23	1	1	7	95.8	87.5
Wu2	2003	China	MRI	Retrospective	23‐75	≥10	Biopsy or follow‐up	32	20	1	4	7	83.3	87.5
Yen1	2003	China	PET	Prospective	16‐75	≥4	Biopsy or follow‐up	67	21	3	0	43	100.0	93.5
Yen2	2003	China	PET	Prospective	16‐75	≥4	Biopsy or follow‐up	67	13	26	8	20	61.9	43.5
Tsai	2003	China	PET	Prospective	—	≥4	Biopsy	20	7	1	0	12	100.0	92.3
Weng	2003	China	PET	Prospective	36‐60	≥4	Biopsy or follow‐up	26	12	0	1	13	92.3	100.0
Tai	2003	China	MRI	Prospective	19‐75	≥4	Follow‐up	26	12	1	1	12	92.3	92.3
Shiau	2003	China	PET	Prospective	22‐75	≥6	Biopsy and follow‐up	30	13	1	2	14	86.7	93.3
Ng	2004	China	PET	Prospective	4‐90	≥4	Follow‐up	37	17	8	2	10	89.5	55.6
Yu	2004	China	PET/CT	Retrospective	26‐71	≥6	Biopsy and follow‐up	38	12	2	0	24	100.0	92.3
Luo	2005	China	PET	Retrospective	26‐72	≥2	Biopsy and follow‐up	75	38	5	2	30	95.0	85.7
Lin	2005	China	PET	Retrospective	45	≥2	Biopsy and follow‐up	28	16	2	2	8	88.9	80.0
Yen	2005	China	PET	Retrospective	16‐75	≥4	Biopsy and follow‐up	64	33	3	3	25	91.7	89.3
Wu	2005	China	PET/CT	Retrospective	—	≥2.5	Biopsy and follow‐up	36	11	2	2	21	84.6	91.3
Chan1	2006	China	PET	Prospective	30‐83	≥3	Biopsy and follow‐up	34	21	2	1	10	95.5	83.3
Chan2	2006	China	PET	Prospective	30‐83	≥3	Biopsy and follow‐up	112	3	6	1	102	75.0	94.4
Chan3	2006	China	MRI	Prospective	30‐83	≥3	Biopsy and follow‐up	34	21	3	1	9	95.5	75.0
Chan4	2006	China	MRI	Prospective	30‐83	≥3	Biopsy and follow‐up	112	3	11	1	97	75.0	89.8
Wu	2006	China	MRI	Prospective	27‐69	—	Biopsy and follow‐up	78	35	13	3	27	92.1	67.5
Xiao	2007	China	PET/CT	Retrospective	27‐59	≥3	Biopsy and follow‐up	20	14	1	1	4	93.3	80.0
Shen	2007	China	PET/CT	Retrospective	35‐79	≥3	Biopsy and follow‐up	15	6	1	1	7	85.7	87.5
Pang	2007	China	PET/CT	Retrospective	27	≥3	Biopsy	27	18	2	1	6	94.7	75.0
Li	2007	China	PET	Retrospective	46	≥3	Biopsy	41	22	13	6	15	78.6	53.6
Xue	2007	China	MRI	Retrospective	24‐73	≥3	Biopsy	63	18	9	6	30	75.0	76.9
Comoretto	2008	Italy	PET/CT	Retrospective	17‐19	≥2	Biopsy and follow‐up	63	27	4	1	31	96.4	88.6
Yen	2009	China	PET/CT	Retrospective	35‐68	≥4	Biopsy and follow‐up	27	10	0	5	12	66.7	100.0
Al Amro	2009	Saudi Arabia	PET	Retrospective	13‐80	≥2	Biopsy and follow‐up	55	11	2	0	42	100.0	95.5
Zhang1	2010	China	PET/CT	Retrospective	31‐62	≥6	Biopsy and follow‐up	21	12	0	1	8	92.3	100.0
Zhang2	2010	China	MRI	Retrospective	31‐62	≥6	Biopsy and follow‐up	21	13	1	0	7	100.0	87.5
He	2010	China	PET/CT	Retrospective	3‐36	≥8	Biopsy and follow‐up	20	8	1	0	11	100.0	91.7
Ng	2010	China	MRI	Prospective	19‐84	≥6	Biopsy and follow‐up	179	25	7	4	173	86.2	96.1
Ng	2010	China	PET/CT	Prospective	19‐84	≥6	Biopsy and follow‐up	179	25	6	4	144	86.2	96.0
Huang	2012	China	PET/CT	Prospective	19‐77	≥6	Biopsy and follow‐up	70	11	1	1	57	91.7	98.3
Ma1	2013	China	PET/CT	Prospective	—	≥6	Biopsy and follow‐up	48	30	9	4	5	88.2	35.7
Ma2	2013	China	MRI	Prospective	—	≥6	Biopsy and follow‐up	48	32	8	2	6	94.1	42.9
Ma3	2013	China	PET/CT	Prospective	19‐83	≥6	Biopsy and follow‐up	89	67	4	5	13	93.1	76.5
Ma4	2013	China	MRI	Prospective	19‐83	≥6	Biopsy and follow‐up	89	60	3	12	14	83.3	82.4
Lin1	2013	China	MRI	Prospective	9‐76	≥6	Biopsy and follow‐up	108	10	44	3	51	76.9	53.7
Lin2	2013	China	MRI	Prospective	9‐76	≥6	Biopsy and follow‐up	108	3	15	10	80	23.1	84.2
Lin3	2013	China	MRI	Prospective	9‐76	≥6	Biopsy and follow‐up	108	2	47	11	48	15.4	50.5
Zhou1	2014	China	MRI	Retrospective	23‐75	≥12	Follow‐up	37	15	1	1	21	93.8	95.5
Zhou2	2014	China	PET/CT	Retrospective	23‐75	≥12	Follow‐up	37	14	2	2	21	87.5	91.3
Lu1	2014	China	PET/CT	Retrospective	22‐79	≥6	Follow‐up	57	42	4	1	10	97.7	71.4
Lu2	2014	China	MRI	Retrospective	22‐79	≥6	Follow‐up	57	35	6	8	8	81.4	57.1
Tian	2014	China	PET/CT	Retrospective	17‐75	≥6	Follow‐up	89	59	5	0	25	100.0	83.3
Wang	2014	China	MRI	Retrospective	21‐82	≥3	Biopsy and follow‐up	90	28	13	11	38	71.8	74.5
Liang1	2015	China	PET/CT	Retrospective	18‐80	≥1	Biopsy and follow‐up	55	22	0	4	29	84.6	100.0
Liang2	2015	China	PET/CT	Retrospective	18‐80	≥1	Biopsy and follow‐up	31	13	2	6	10	68.4	83.3
Li	2015	China	PET/CT	Retrospective	—	≥6	Biopsy	54	11	1	0	42	100.0	97.7
Hei1	2016	China	CT	Retrospective	18‐72	≥6	Biopsy and follow‐up	63	21	11	9	22	70.0	66.7
Hei2	2016	China	MRI	Retrospective	18‐72	≥6	Biopsy and follow‐up	63	25	6	5	27	83.3	81.8

### 3.3 Assessment of quality

Data S3 and S4 summarized the details of risk of bias. Overall, the whole quality of the included studies was pretty good. The proportion of high‐risk bias studies was very low. The main issue was flow and timing (unclear if there was an appropriate interval between index test and reference standard). Totally, five and 24 studies were categorized as low and unclear risk of bias, respectively, because of flow and timing. Two studies were categorized as unclear risk of bias in index test and three studies as unclear risk of bias in reference standard.

### 3.4 Pooled results

**Figure 2:**
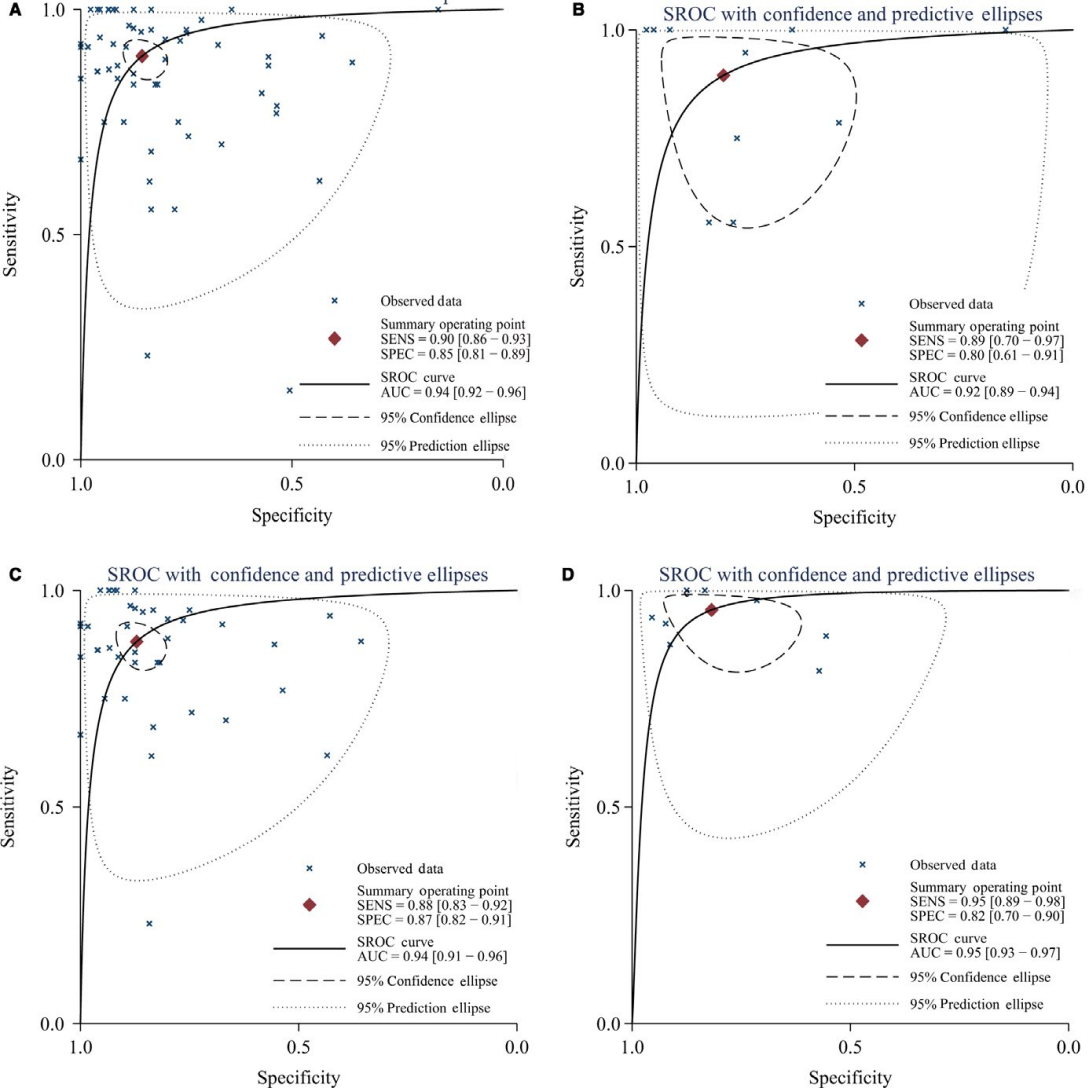
The SROC curve of PET/CT and MRI for local recurrence and residue of nasopharyngeal carcinoma (A, all studies; B, biopsy alone; C, biopsy and follow‐up; D, follow‐up alone)

**Figure 3:**
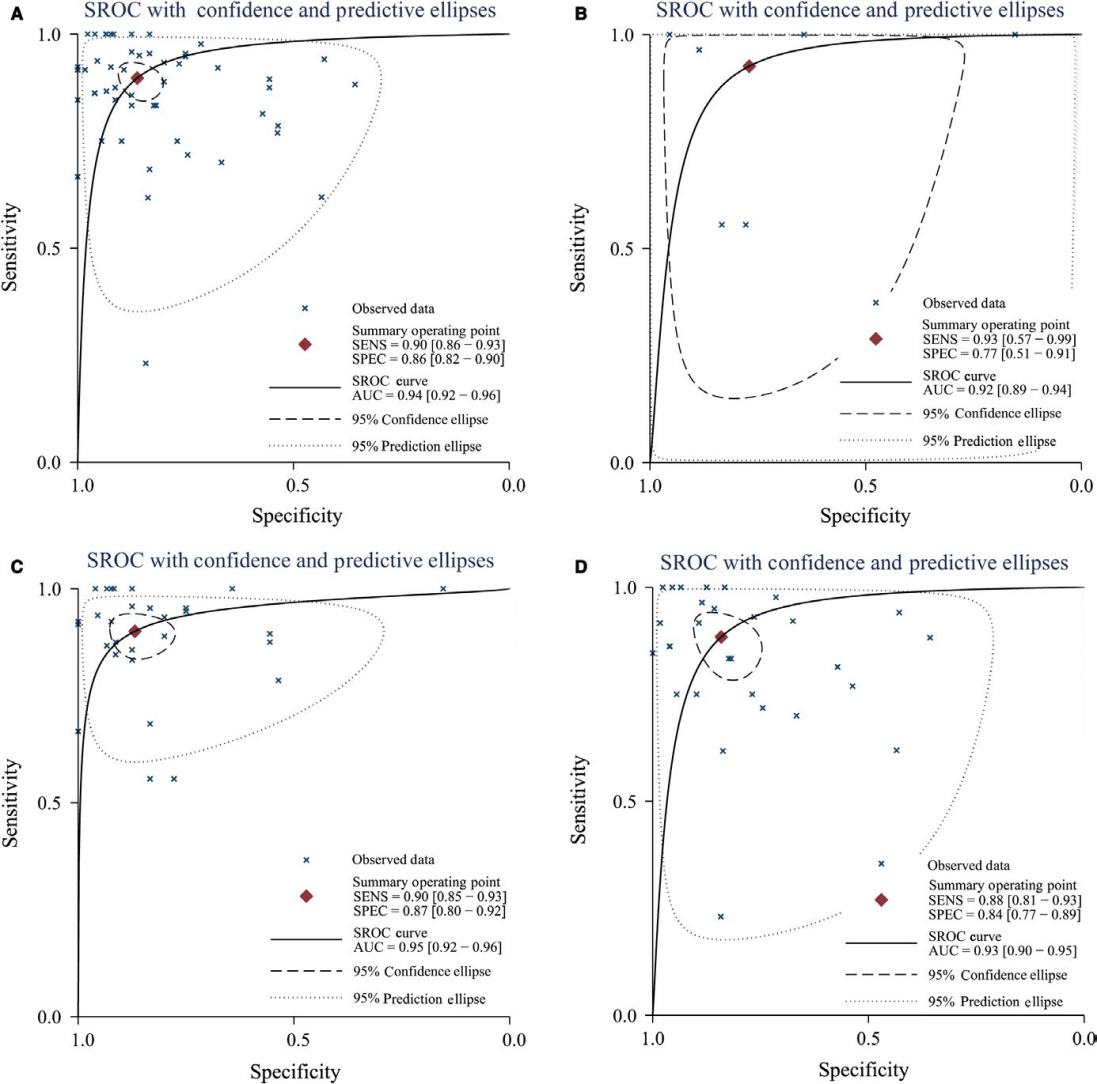
The SROC curve of PET/CT and MRI for local recurrence and residue of nasopharyngeal carcinoma (A, China; B, other country; C, sample size≤45; D, sample size>45)

**Figure 4:**
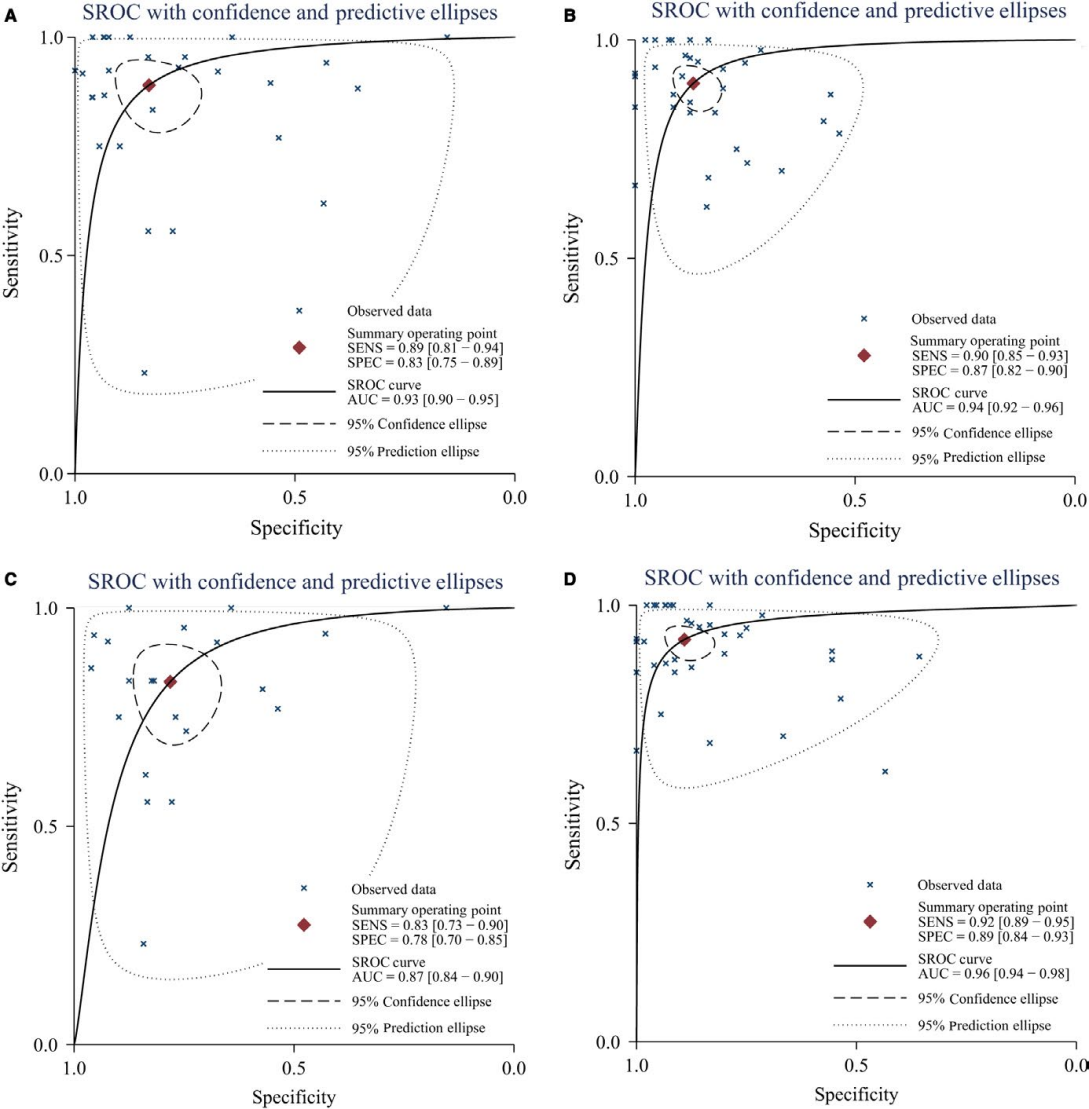
The SROC curve of PET/CT and MRI for local recurrence and residue of nasopharyngeal carcinoma (A, prospective; B, retrospective; C, MRI alone; D, PET/CT alone)

The estimated results about the diagnostic ability of PET‐CT/MRI for local recurrent and residual NPC were shown in Table 2. The random‐effect models were used because of the high heterogeneity (*I* ^2^ > 50%). The other overall estimated results were as follows: sensitivity = 0.90 [95% CI: 0.86‐0.93, Figure 2], specificity = 0.85 [95% CI: 0.81‐0.89, Figure 3], PLR = 5.57 [95% CI: 3.74‐8.31], NLR = 0.18 [95% CI: 0.11‐0.28], and DOR = 31.33 [95% CI: 15.19‐64.61]. In addition, the pooled area under the curve (AUC) of PET‐CT/MRI was 0.94 [95% CI: 0.92‐0.96, Figure 4A], which indicated a high diagnostic ability.

**Table 2:** Summary estimated of diagnostic performance of MRI/PET/CT for residual/recurrent nasopharyngeal carcinoma

Category	Number of study data	SEN (95% CI)	SPE (95% CI)	PLR (95% CI)	NLR (95% CI)	DOR (95% CI)	AUC (95% CI)
Overall		0.90 [0.86‐0.93]	0.85 [0.81‐0.89]	5.57 [3.74‐8.31]	0.18 [0.11‐0.28]	31.33 [15.19‐64.61]	0.94 [0.92‐0.96]
Golden standard							
Biopsy	10	0.89 [0.70‐0.97]	0.80 [0.61‐0.91]	4.48 [2.06‐9.76]	0.13 [0.04‐0.44]	34.12 [5.95‐195.64]	0.92 [0.89‐0.94]
Biopsy and follow‐up	43	0.88 [0.83‐0.92]	0.87 [0.82‐0.91]	6.85 [4.79‐9.78]	0.14 [0.09‐0.20]	3.92 [3.28‐4.55]	0.94 [0.91‐0.96]
Follow‐up	8	0.96 [0.89‐0.98]	0.82 [0.70‐0.90]	5.25 [3.06‐9.01]	0.06 [0.02‐0.15]	94.59 [24.87‐359.77]	0.95 [0.93‐0.97]
Population							
China	55	0.90 [0.86‐0.93]	0.86 [0.82‐0.90]	6.50 [4.80‐8.81]	0.12 [0.08‐0.17]	54.49 [30.84‐96.25]	0.94 [0.92‐0.96]
Others	6	0.93 [0.57‐0.99]	0.77 [0.51‐0.91]	4.03 [1.65‐9.80]	0.10 [0.01‐0.76]	41.92 [3.71‐473.39]	0.92 [0.89‐0.94]
Sample size							
≤45	31	0.90 [0.85‐0.93]	0.87 [0.80‐0.92]	6.84 [4.42‐10.58]	0.12 [0.08‐0.17]	59.681 [29.73‐119.82]	0.95 [0.92‐0.96]
>45	30	0.88 [0.81‐0.93]	0.84 [0.77‐0.89]	5.57 [3.73‐8.31]	0.14 [0.08‐0.23]	40.35 [17.66‐92.15]	0.93 [0.90‐0.95]
Study design							
Prospective	29	0.89 [0.81‐0.94]	0.83 [0.75‐0.89]	3.75 [2.69‐5.24]	0.16 [0.09‐0.28]	27.03 [10.78‐67.75]	0.93 [0.90‐0.95]
Retrospective	32	0.90 [0.85‐0.93]	0.87 [0.82‐0.90]	6.81 [4.89‐9.48]	0.12 [0.08‐0.17]	59.37 [31.22‐112.90]	0.94 [0.92‐0.96]
Examination methods							
MRI	23	0.83 [0.72‐0.90]	0.78 [0.70‐0.85]	3.79 [2.64‐5.47]	0.22 [0.13‐0.36]	17.55 [8.11‐37.97]	0.87 [0.84‐0.90]
PET/CT	38	0.92 [0.89‐0.95]	0.89 [0.84‐0.93]	8.46 [5.67‐12.62]	0.09 [0.06‐0.13]	95.50 [49.17‐185.47]	0.96 [0.94‐0.98]

**Figure 5:**
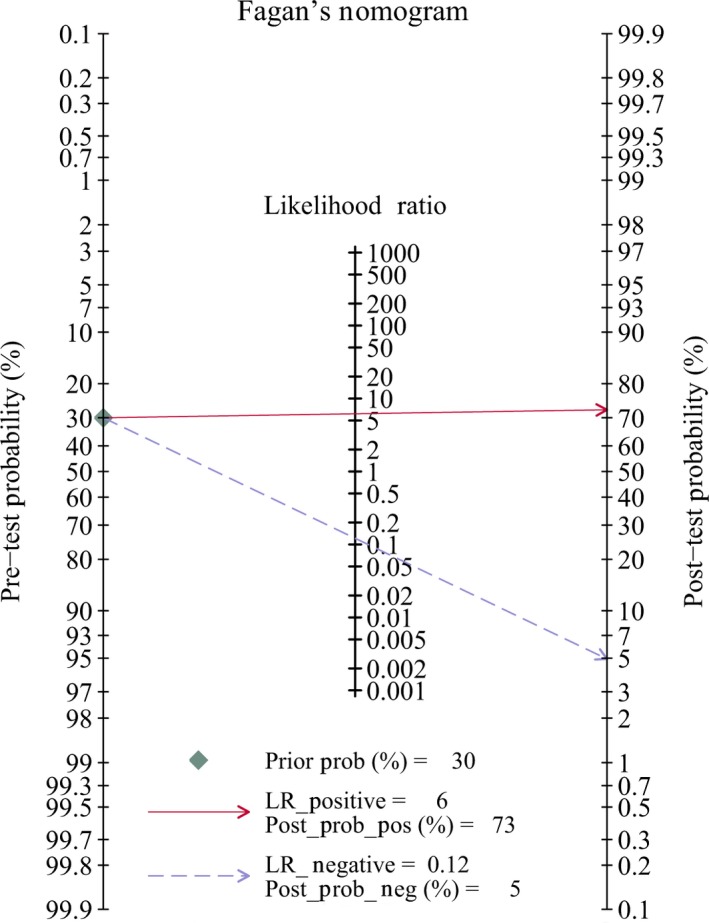
Fagan diagram assessing the overall diagnostic value of PET/CT and MRI for local recurrence and residue of nasopharyngeal carcinoma

Table 1 also presented the results of subgroup analyses by population (China vs other countries), sample size (≤45 vs >45), examination methods (MRI vs PET/CT), study design (prospective vs retrospective), and golden standard (biopsy, follow‐up, or both). No significant difference was found in different standards, populations, sample sizes, or study designs. Similar sensitivity and specificity were found among different subgroups. Similar pooled AUCs were found in golden standards (Figure 4), populations (Figure 5), sample size (Figure 5), or study design, indicating these factors were not the decisive factors of heterogeneity. However, significant difference was found between examination methods. MRI vs PET‐CT had lower sensitivity (0.83 [95% CI: 0.72‐0.90] vs 0.92 [0.89‐0.95]) and specificity (0.78 [0.70‐0.85] vs 0.89 [0.84‐0.93]). PET‐CT showed better PLR, NLR, and DOR than MRI (Table 2). The AUCs of MRI and PET‐CT were 0.87 and 0.96, respectively. No‐cross of 95% CI was found between MRI and PET/CT (0.87‐0.90 vs 0.94‐0.98). The pooled sensitivity and specificity forest plots and Fagan's Nomogram of the examination methods were presented in Data S5. The overall Fagan's Nomogram was presented in Figure 5. If the pre‐test probability was 30%, the post‐test probability would reach about 73% with PLR of 6.

### 3.5 Meta‐regression analysis

Considering the high heterogeneity within studies, we conducted the meta‐regression to explore the potential influencing factors. The meta‐regression results indicated the examination method (PET/CT vs MRI) was a potential source of heterogeneity (*P < *0.001; Table 3). The subgroup analysis showed the diagnostic ability of MRI was slightly weaker than PET/CT.

**Table 3:** Meta‐analysis regression of estimating covariate effect

Parameters	*I*‐squared (95% CI)	LRT chi‐square	*P *value
Publication year	0.00 [0.00‐100.00]	1.24	0.540
Country	0.00 [0.00‐100.00]	1.29	0.52
Examination methods	82.66 [63.28‐100.00]	11.54	0.00
Study design	0.00 [0.00‐100.00]	1.62	0.45
Golden standard	2.38 [0.00‐100.00]	2.05	0.36
Sample size	72.19 [38.34‐100.00]	7.19	0.03

### 3.6 Publication bias

The publication bias was assessed by Deek's line regression plot. The X‐ and Y‐directions were effective sample size and diagnostic odds ratio, respectively. The angle between the regression line and the X‐direction was close to zero, which means no publication bias (*P* = 0.954, Data S6). The regression line was almost parallel with the X‐direction. Begg's test did not indicate publication bias (*Z* = 1.200, *P* = 0.230), indicating the publication bias of the current study was limited, but Egger's test showed some publication bias (*t* = 5.430, *P < *0.001).

## 4 DISCUSSION

PET/CT and MRI both show relatively high overall accuracy in diagnosing local recurrence and residue of NPC, but PET‐CT is superior over MRI according to the subgroup analyses. Meta‐regression suggests the examination method is the main source of heterogeneity. This is the largest study so far that presents more accurate estimation about PET‐CT and MRI in diagnosing recurrent and residual NPC. Two other studies also compared 18F‐FDG PET/CT, MRI, and single‐photon emission computed tomography (SPECT) in diagnosing local residual/recurrent NPC,^20^, ^21^ but these studies had several limitations. First, their results were reported in 2007 and 2016, respectively, but the search period was from 1990 to 2014 after which many new studies were reported. Our study includes 27 new studies. Though Wei's report included 17 studies, only <10 studies were focused on MRI or PET/CT. Second, our subgroup analysis by the gold standard (biopsy, follow‐up, or both) showed no significant diagnostic differences, which excluded the verification bias mentioned by the two studies. Third, though they reported PET/CT and SPECT were superior over MRI in distinguishing recurrent NPC from fibrosis after radiotherapy, the supplementary data indicated the SROCs of SPECT and MRI overlapped, which means the significant difference was doubtful. Finally, the two studies and the present study all found high heterogeneity, but our meta‐regression analysis identified the examination method as one of the heterogeneity sources. Moreover, the latest version of Assessment of Methodological Quality was used in the present study.

Whether there is local residue or recurrence is extremely important for NPC staging and treatment plan. As reported, NPC patients with local residue had poorer prognosis and higher risk of recurrence.^22^ MRI was previously considered as the golden standard of local therapy efficacy in NPC.^23^ However, the inflammatory changes after radiotherapy interfered the image interpretation and lowered the specificity (range from 44% to 83%).^24^ On the contrary, PET/CT shows strong diagnostic ability of efficacy evaluation and lesion distinguishing (specificity: 93.4%). Some studies compared PET/CT and MRI in distinguishing residual/recurrent NPC, but the results were inconsistent.^25^ Most studies reported PET/CT was superior over MRI in diagnosing local recurrence and residue of NPC.^26^, ^27^, ^28^ However, a retrospective study involving 63 consecutive patients showed MRI vs PET/CT had slightly, but not significantly, higher overall accuracy in diagnosing residual and/or recurrent NPC (92.1% vs 85.7%).^29^ This difference from other studies may be attributed to the overestimated overall diagnostic accuracy due to the small sample size. Our results with a larger sample indicated PET/CT vs MRI showed higher overall diagnostic accuracy with sensitivity (92% vs 83%), specificity (89% vs 78%), and SROC (0.96 vs 0.87).

The differences of overall accuracy between PET/CT and MRI may be attributed to the imaging principle. It is generally agreed that MRI outperforms CT in detecting residual and recurrent NPC.^30^ MRI can efficiently distinguish tumor lesions from normal tissues and identify the fibrosis and tumor recurrence after local radiotherapy. The tissue‐specific signals of MRI clearly outline the scope, size, and depth of tumor invasion and localize the nasopharyngeal mass, involved areas (especially the parapharyngeal space), perineural skull infiltration, skull damage, and intracranial invasion. With the wide clinical application, MRI has become an important method for the pretreatment examination and post‐radiotherapy efficacy judgment of NPC. However, MRI still has limitations in identifying the swollen lymph nodes, since the diagnosis is dependent on the lymph nodes size. The pathology patterns of lymph nodes are unclear, which may lead to misdiagnosis or missed diagnosis of diseases. Different from MRI, the 18F‐FDG PET/CT with unique metabolic imaging features can more correctly diagnose lymph node properties.

PET/CT has higher overall diagnostic accuracy for recurrent NPC and generally consists of a PET scanner, a high‐resolution spiral CT scanner, and an operating system that will combine two types of scan images. PET and CT can be obtained simultaneously with one scan. PET/CT images combine the metabolic imaging characteristics of PET scanners with the anatomical imaging characteristics of CT scanners, which make up for the unclear positioning of PET and solve the low accuracy of CT. Given the biological characteristics of specific tumor tissues and the imaging characteristics of PET/CT, PET/CT has significant advantages in differentiating post‐radiotherapy NPC from fibrosis and tumor local recurrence or lymph node metastasis. Currently, the most commonly used nuclide tracer is 18F‐FDG, imaging of which distinguishes benign and malignancy mainly according to the difference in glucose metabolism between normal tissues and tumor tissues in the human body. The principle is that SPECT 18F‐FDG after entering human malignant tumor cells is decomposed by hexokinase into an undecomposed 6‐phosphoric acid deoxidizing glucose, which largely accumulates in the tumor cells and significantly increases the metabolism activity of tumor tissues and uptake of 18F‐FDG. However, as a tumor‐nonspecific imaging agent, the uptake of 18F‐FDG in the irradiation area can also be increased by inflammatory changes.^31^ Therefore, PET/CT contains some false positives and false negatives.

The present study has several limitations. First, the heterogeneity within studies is quite high, which was addressed here by two ways. The subgroup analysis only by the golden standard found the source of heterogeneity, but not population, study design, examination methods, or sample size. Only significant difference of overall accuracy was found. Then, multivariate meta‐regression including the above factors indicated the examination method may be associated with heterogeneity. Second, some factors and unmeasured or unreported study characteristics such age gender and stage cannot be obtained for further subgroup, which may overestimate or underestimate the overall pooled results. The reason is that the sample size was too small to further subgroup analysis in each study. Third, the golden standard was mixed (biopsy, follow‐up, or both), but biopsy would be better. However, the subgroup analysis did not indicate significant difference among three types. Moreover, MRI or PET ∓CT had enormously evolved during the long search period from 1991 to 2018. However, the meta‐regression indicated publication year seemingly had no effect on the estimated covariate effect.

In conclusion, PET/CT and MRI both show quite high overall diagnostic ability for local recurrence/residue of NPC. But the subgroup analyses indicate PET‐CT is superior over MRI in diagnosis of local recurrent and residual NPC after radiotherapy. The examination methods affect the heterogeneity within studies. The present study provides stronger evidence for clinical practice.

## CONFLICT OF INTEREST

The authors declare that they have no conflict of interest.
