# Supplementary material for: Positron emission tomography/computed tomography outperforms MRI in the diagnosis of local recurrence and residue of nasopharyngeal carcinoma: An update evidence from 44 studies
Source: Cancer Med. 2018 Dec 21;8(1):67–79. doi: 10.1002/cam4.1882 (PMC6346220; doi:10.1002/cam4.1882)
Supplement: Supplementary file 2 [file CAM4-8-67-s002.docx]

**Supplementary material 1: References lists of study included in the meta-analysis**

1.Al-Amro A, Saleem M, Bakheet S et al (2009) The Role of 18-FDG Positron Emission Tomography (FDG-PET) in Detecting Post- Radiotherapy Loco Regional Relapse/Residual Disease in Nasopharyngeal Cancer. J Egypt Natl Canc Inst. 21:279-285

2.Chan SC, Ng SH, Chang JT et al (2006) Advantages and pitfalls of 18F-fluoro-2-deoxy-D-glucose positron emission tomography in detecting locally residual or recurrent nasopharyngeal carcinoma: comparison with magnetic resonance imaging. Eur J Nucl Med Mol Imaging. 33:1032-1040

3.Chen YR, Gu XM, Li WX, Pan Y (2002) Detection of residue of nasopharyngeal carcinoma with FDG PET. Chin J Cancer. 21:651-653

4.Chong VF, Fan YF (1997) Detection of recurrent nasopharyngeal carcinoma: MR imaging versus CT. Radiology. 202:463-470

5.Comoretto M, Balestreri L, Borsatti E, Cimitan M, Franchin G, Lise M (2008) Detection and restaging of residual and/or recurrent nasopharyngeal carcinoma after chemotherapy and radiation therapy: comparison of MR imaging and FDG PET/CT. Radiology. 249:203-211

6.Gong QY, Zheng GL, Zhu HY (1991) MRI differentiation of recurrent nasopharyngeal carcinoma from postradiation fibrosis. Comput Med Imaging Graph. 15:423-429

7.He CC, Qu BG, Si YF, Jiang H, Wu JH (2010) Comparison analysis between and FDG-PET/CT and CT in nasopharyngeal carcinoma patients after treatment. Chin J Clinicians. 4:2295-2297

8.Hei ZL, Wang Y, Chen JJ, Guo YH (2016) Discussion on the value of CT and MRI in the diagnosis of recurrence after radiotherapy of nasopharyngeal carcinoma. Continuing Med Edu. 30:147-148

9.Huang SC, Qin ZJ, Ma JQ, Wei XL, Yan LM (2012) Diagnositc value of (18)F-FDG PET/CT in residual/ recurrent of nasopharyngeal carcinoma after therapy. Guangxi Med. 34:287-289

10. Jiang SX, Mao MW, Zhao JN (2000) CT and MRI diagnosis of local recurrences or residues after radiotherapy of naopharyngeal carecinoma. J Prac Med Imaging. 1:30-33

11. Kao CH, Shiau YC, Shen YY, Yen RF (2002) Detection of recurrent or persistent nasopharyngeal carcinomas after radiotherapy with technetium-99m methoxyisobutylisonitrile single photon emission computed tomography and computed tomography: comparison with 18-fluoro-2-deoxyglucose positron emission tomography. Cancer. 94:1981-1986

12. Kostakoglu L, Uysal U, Ozyar E et al (1997) Monitoring response to therapy with thallium-201 and technetium-99m-sestamibi SPECT in nasopharyngeal carcinoma. J Nucl Med. 38:1009-1014

13. Li JP, Zou YJ, Bi W, Fan XM, Zheng XL, Lu JS (2015) Value of dynamic contrast-enhanced MRI and F-FDG PET-CT in differentiating recurrent nasophyarygeal carcinoma. Diag Imaging & Inter Radio. 24:471-475

14. Li WH, Huang XM, Zeng L et al (2007) Diagnositc value of (18)F-FDG PET/CT in residual/ recurrent of nasopharyngeal carcinoma after radiotherapy. Chin J Otorhinolaryngol Head Neck Surg. 42:65-66

15. Liang Y, Wu Y, Geng JH et al (2015) 18F-FDG PET/CT for detection of residual/recurrent nasopharyngeal carcinoma after therapy. Chin Med Equi. 12:66-70

16. Lin GW, Wang LX, Ji M, Qian HZ (2013) The use of MR imaging to detect residual versus recurrent nasopharyngeal carcinoma following treatment with radiation therapy. Eur J Radiol. 82:2240-2246

17. Lin YR, Chen YR, Li WX (2005) Diagnostic value of FDG-PET for recurrence in patients with nasopharyngeal carcinoma. Prac Med J. 21:48-49

18. Lu Y (2014) The application value of 18F-FDG PET/CT in early detecting the recurrence of nasopharyngeal carcinoma after radiotherapy., vol 硕士. Soochow University, p 33

19. Luo YW, Tang AW, Zhu YQ, Chen LG, Chen YR (2005) Diagnostic value of 18F-FDG PET for recurrence and residue in nasopharyngeal carcinoma patients. Chin J Nucl Med. 5:55

20. M M, Du XD (2013) PET-CT and EBV-DNA in preiction of the recurrence of nasopharyngeal carcinoma. Chin Arch Otolaryngol Head Neck Surg. 20:21-23

21. Ma XM, Ye M, Chen HY, Bai YR, Xiao XS (2013) Diagnostic value of magnetic resonance imaging and positron emission Tomography in nasopharyngeal carcinoma post-radiation with skull base recurrence. J Chin Oncol. 19:175-178

22. Ng SH, Chan SC, Yen TC et al (2010) Comprehensive imaging of residual/ recurrent nasopharyngeal carcinoma using whole-body MRI at 3 T compared with FDG-PET-CT. Eur Radiol. 20:2229-2240

23. Ng SH, Joseph CT, Chan SC et al (2004) Clinical usefulness of 18F-FDG PET in nasopharyngeal carcinoma patients with questionable MRI findings for recurrence. J Nucl Med. 45:1669-1676

24. Pang QS, Wang J, Dai D, Zhu YJ, Wang P (2007) Value of 18FDG PET/CT after radiotherapy in nasopharyngeal carcinoma. Chin J Radiat Oncol. 16:411-415

25. Peng N, Yen S, Liu W, Tsay D, Liu R (2000) Evaluation of the Effect of Radiation Therapy to Nasopharyngeal Carcinoma by Positron Emission Tomography with 2-. Clin Positron Imaging. 3:51-56

26. Shen C, Liu CY, Yao SZ (2007) Application of 18F-FDG PET/CT in follow-up of naspharyngeal carcinoma after treatment. J Med Imaging. 17:674-676

27. Shiau YC, Liu FY, Huang WS, Yen RF, Kao CH (2003) Using thallium-201 SPECT to detect recurrent or residual nasopharyngeal carcinoma after radiotherapy in patients with indeterminate CT findings. Head Neck. 25:645-648

28. Tai CJ, Liang JA, Yang SN, Tsai MH, Lin CC, Kao CH (2003) Detection of recurrent nasopharyngeal carcinomas with thallium-201 single-photon emission computed tomography in patients with indeterminate magnetic resonance imaging findings after radiotherapy. Head Neck. 25:227-231

29. Tian YL (2014) Diagnostic and prognositc value of 18-F-FDG PET/CT for nasopharyngeal carcinoma patients follo-up after comprehensive therapy., vol 博士. Huangzhonh University of Science and Technology, p 78

30. Tsai MH, Huang WS, Tsai JJ, Chen YK, Changlai SP, Kao CH (2003) Differentiating recurrent or residual nasopharyngeal carcinomas from post-radiotherapy changes with 18-fluoro-2-deoxyglucose positron emission tomography and thallium-201 single photon emission computed tomography in patients with indeterminate computed tomography findings. Anticancer Res. 23:3513-3516

31. Tsai MH, Shiau YC, Kao CH, Shen YY, Lin CC, Lee CC (2002) Detection of recurrent nasopharyngeal carcinomas with positron emission tomography using 18-fluoro-2-deoxyglucose in patients with indeterminate magnetic resonance imaging findings after radiotherapy. J Cancer Res Clin Oncol. 128:279-282

32. Wang C (2014) Study on diagnostic value of diffusioin weighted MRI on local recurrence of nasopharygeal carcinoma., vol 硕士. Guangxi Medical University, p 98

33. Weng YC, Yen RF, Tsai MH, Wang JJ, Ho ST, Kao CH (2003) Detection of recurrent nasopharyngeal carcinomas after radiotherapy with technetium-99m tetrofosmin single photon emission computed tomography in patients with indeterminate magnetic resonance imaging findings. Cancer Invest. 21:695-700

34. Wu CF, Deng B, Long SL, Chenb XF, Liang R (2006) The value of nucle in tumor affin in imaging computed tomography and MRI imaging in the diagnosis of recurrent in nasopharyngeal carcinoma after radiotherapy. J Chin Phys. 18:768-770

35. Wu D, Chen L (2003) Clinical value of F-fluoro-2-deoxy-D-glucose positron emission tomography in detecting recurrent nasopharyngeal carcinoma at parapharyngeal space after radiotherapy. J First Mil Med Univ. 2:159-161

36. Wu HB, Wang QS, Wang MF, Wang XL, Guo XJ (2005) The imaging manifestation and clinical value of PET/CT in nasopharyngeal carcinoma. 25:347-349

37. Xiao Y, Li YZ, Guo XJ, Zhou N (2007) Diagnositc value of (18)F-FDG PET/CT in residual/ recurrent of nasopharyngeal carcinoma after radiotherapy. Guangdong Med J. 28:1140-1141

38. Xue JX, Duan RQ, Leng XM, Ma CJ (2007) Evaluation on CT and MRI image features for the diagnosis of local recurrences or residues of nasopharyngeal carcinoma after radiotherapy. Chin Otorhinolaryngol J Integ Med. 15:284-287

39. Yen RF, Hong RL, Tzen KY, Pan MH, Chen TH (2005) Whole-body 18F-FDG PET in recurrent or metastatic nasopharyngeal carcinoma. J Nucl Med. 46:770-774

40. Yen RF, Hung RL, Pan MH et al (2003) 18-fluoro-2-deoxyglucose positron emission tomography in detecting residual/recurrent nasopharyngeal carcinomas and comparison with magnetic resonance imaging. Cancer. 98:283-287

41. Yen RF, Ting LL, Cheng MF, Wu YW, Tzen KY, Hong RL (2009) Usefulness of 201TL SPECT/CT relative to 18F-FDG PET/CT in detecting recurrent skull base nasopharyngeal carcinoma. Head Neck. 31:717-724

42. Yu DF, Zuo ZT, Dai JZ et al (2004) Application of 18F-FDG PET/CT scan in following-up of nasopharyngeal carcinoma after radiotherapy. Chin J Cancer. 23:1538-1541

43. Zhang H, Li GW, Xie AM, Liang ZY (2010) Value of PET/CT and MRI in the diagnosis of recurrence cases after radiotherapy of nasopharyngeal carcinoma. Modern Oncology. 18:2123-2127

44. Zhou MY, Fo M, Wan SH (2014) Clinical value about MRI dynamic enhancement F-FDG PET/CT imaging applied to early diagnosis of recurrent and fibrosis of nasopharynbgeal carcinoma after radiotherapy. Clin Res. 11:34-36
