# Supplementary figures and images for: Positron emission tomography/computed tomography outperforms MRI in the diagnosis of local recurrence and residue of nasopharyngeal carcinoma: An update evidence from 44 studies
Source: Cancer Med. 2018 Dec 21;8(1):67–79. doi: 10.1002/cam4.1882 (PMC6346220; doi:10.1002/cam4.1882)

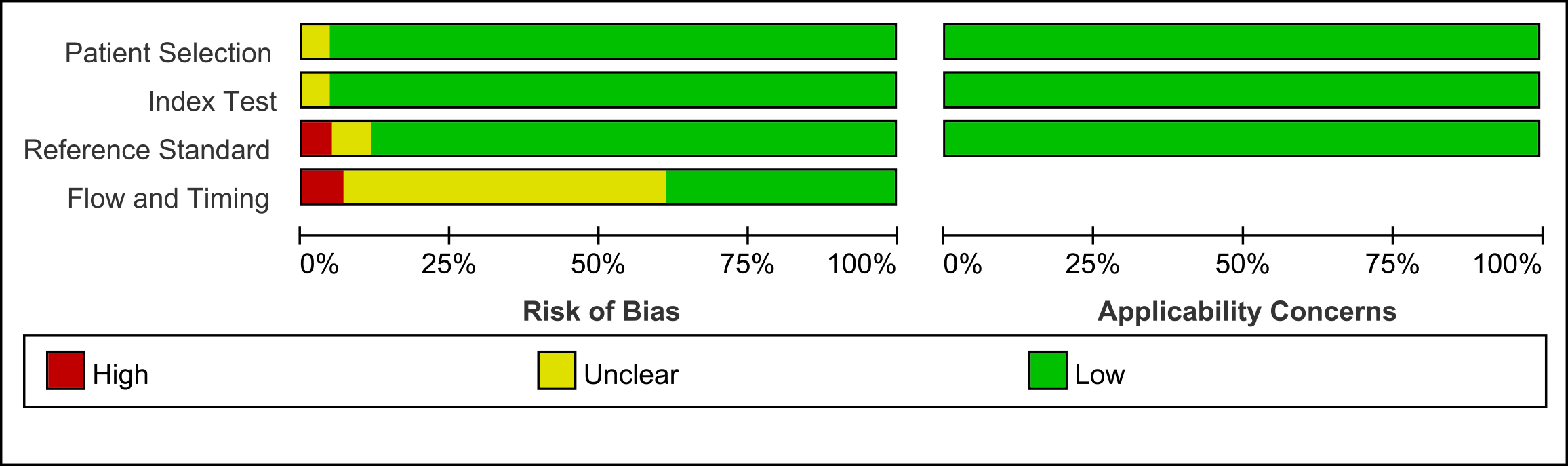

Supplement: Supplementary file 3 [file CAM4-8-67-s003.tif]

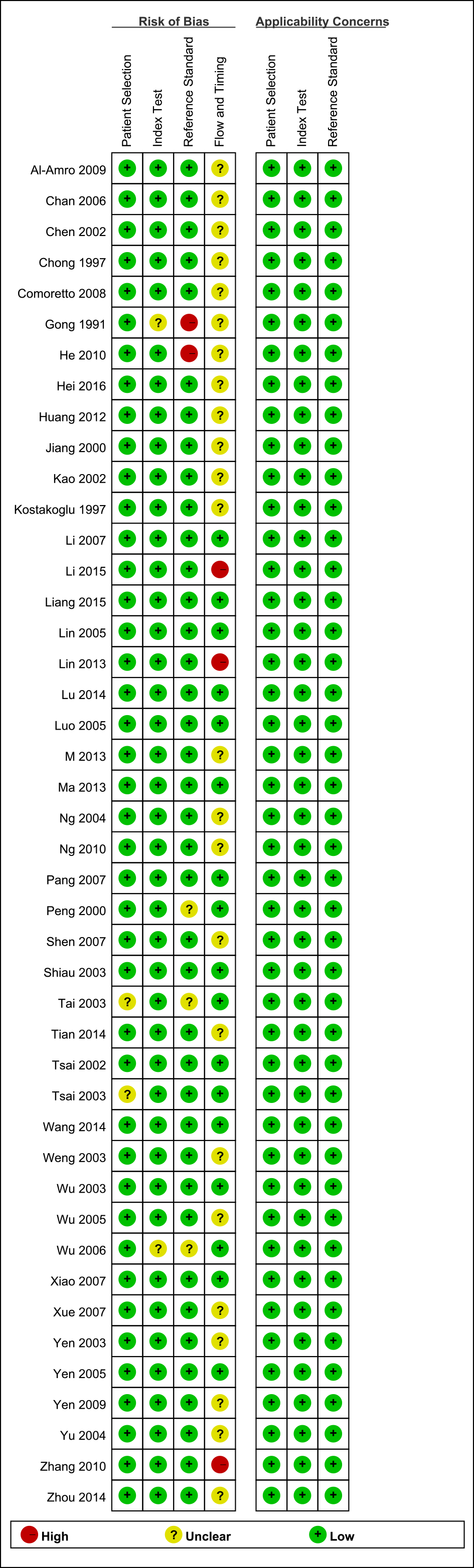

Supplement: Supplementary file 4 [file CAM4-8-67-s004.tif]

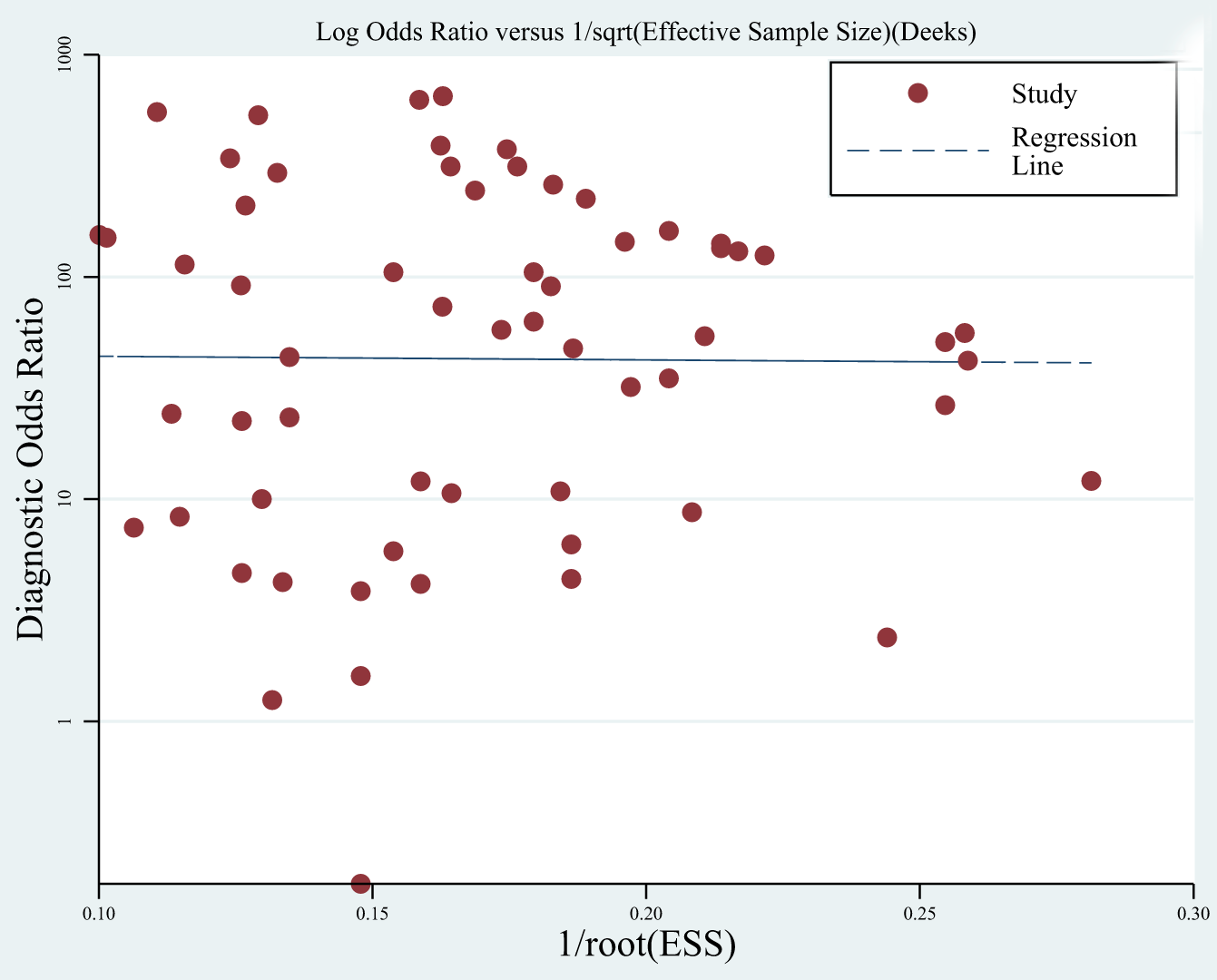

Supplement: Supplementary file 6 [file CAM4-8-67-s006.tif]
