# Supplementary material for: Positron emission tomography/computed tomography outperforms MRI in the diagnosis of local recurrence and residue of nasopharyngeal carcinoma: An update evidence from 44 studies
Source: Cancer Med. 2018 Dec 21;8(1):67–79. doi: 10.1002/cam4.1882 (PMC6346220; doi:10.1002/cam4.1882)
Supplement: Supplementary file 5 [file CAM4-8-67-s005.pdf]

Supplementary material 4 Pooled sensitivity and specificity forest plots and Fagan's Nomogram of PET/CT and MRI

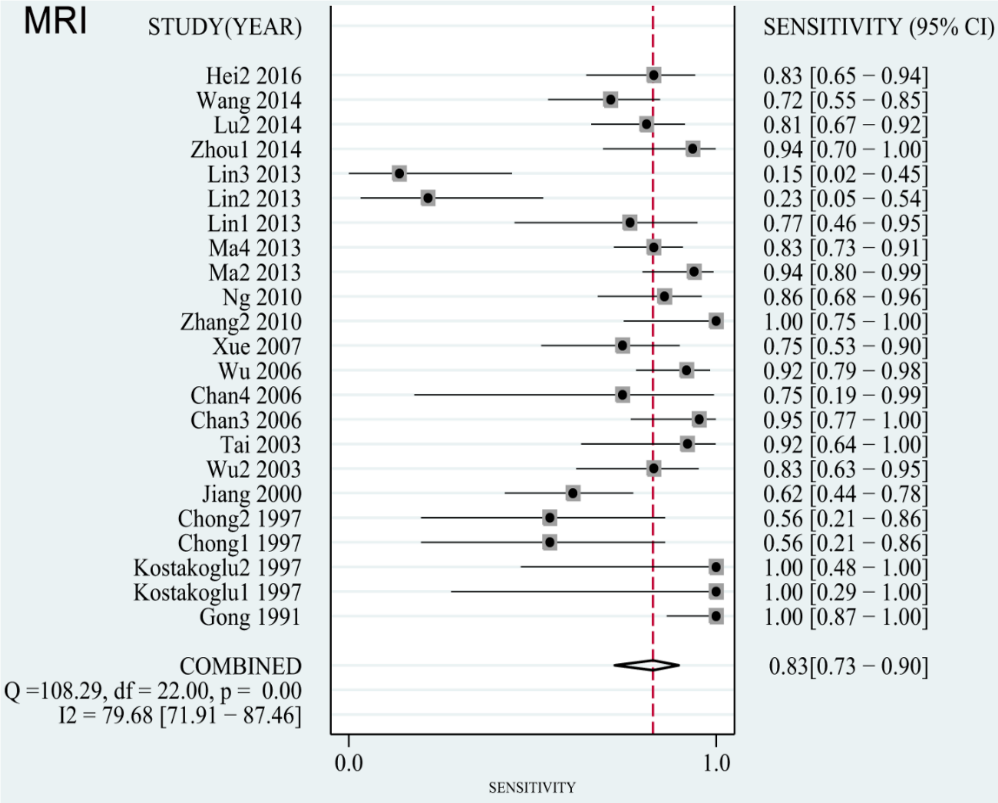

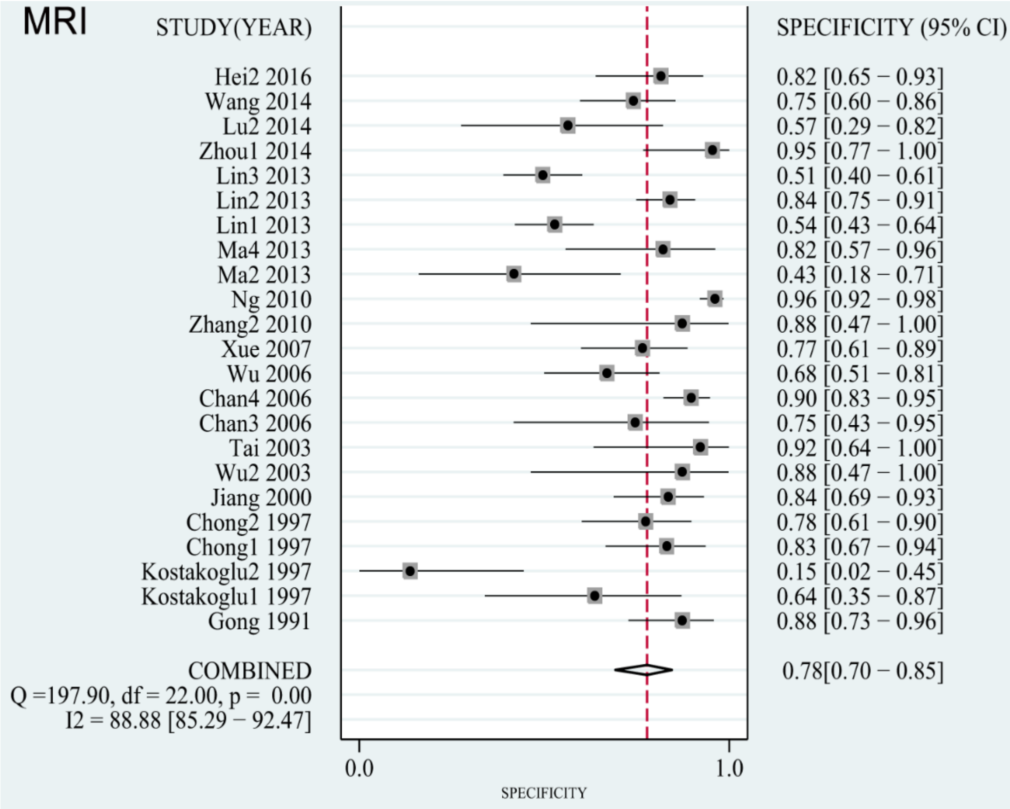

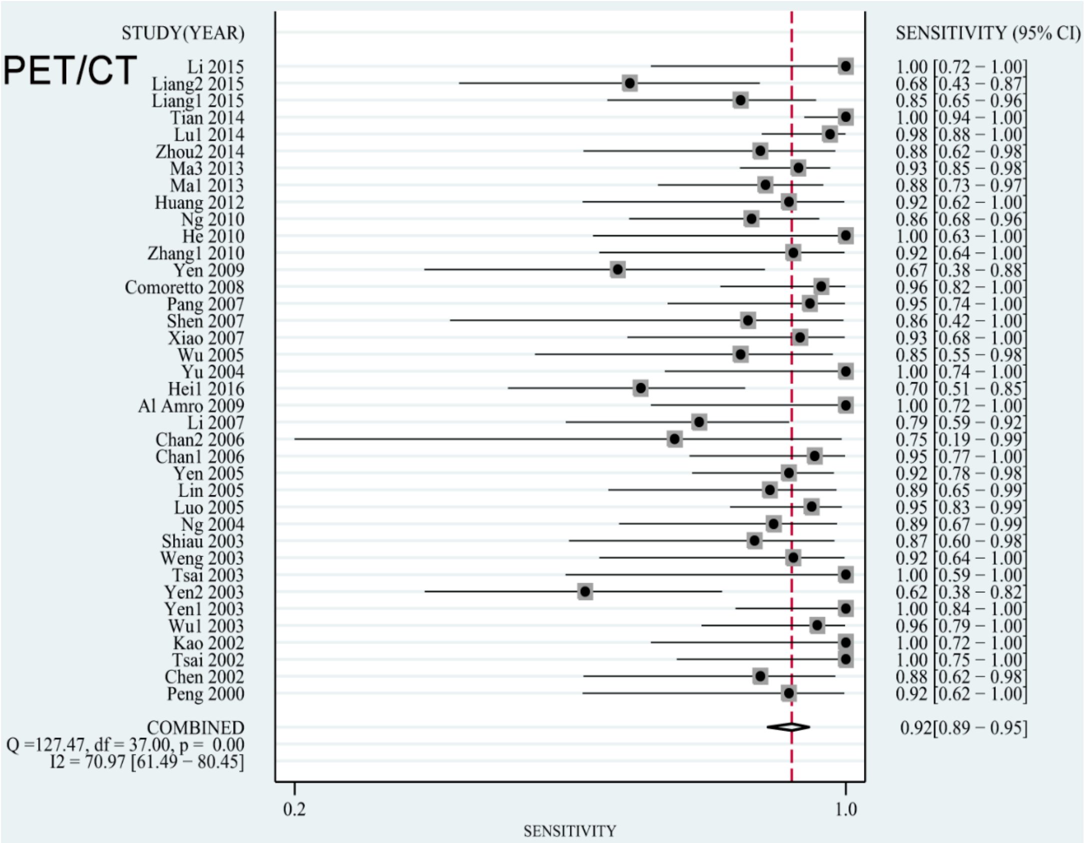
STUDY(YEAR)
PET/CT
Li 2015
Liang2 2015
Liang1 2015
Tian 2014
Lu1 2014
Zhou2 2014
Ma3 2013
Ma1 2013
Huang 2012
Ng 2010
He 2010
Zhang1 2010
Yen 2009
Comoretto 2008
Pang 2007
Shen 2007
Xiao 2007
Wu 2005
Yu 2004
Hei1 2016
Al Amro 2009
Li 2007
Chan2 2006
Chan1 2006
Yen 2005
Lin 2005
Luo 2005
Ng 2004
Shiau 2003
Weng 2003
Tsai 2003
Yen2 2003
Yen1 2003
Wu1 2003
Kao 2002
Tsai 2002
Chen 2002
Peng 2000
COMBINED
Q =127.47, df = 37.00, p = 0.00
I2 = 70.97 [61.49 − 80.45]
SENSITIVITY (95% CI)
1.00 [0.72 − 1.00]
0.68 [0.43 − 0.87]
0.85 [0.65 − 0.96]
1.00 [0.94 − 1.00]
0.98 [0.88 − 1.00]
0.88 [0.62 − 0.98]
0.93 [0.85 − 0.98]
0.88 [0.73 − 0.97]
0.92 [0.62 − 1.00]
0.86 [0.68 − 0.96]
1.00 [0.63 − 1.00]
0.92 [0.64 − 1.00]
0.67 [0.38 − 0.88]
0.96 [0.82 − 1.00]
0.95 [0.74 − 1.00]
0.86 [0.42 − 1.00]
0.93 [0.68 − 1.00]
0.85 [0.55 − 0.98]
1.00 [0.74 − 1.00]
0.70 [0.51 − 0.85]
1.00 [0.72 − 1.00]
0.79 [0.59 − 0.92]
0.75 [0.19 − 0.99]
0.95 [0.77 − 1.00]
0.92 [0.78 − 0.98]
0.89 [0.65 − 0.99]
0.95 [0.83 − 0.99]
0.89 [0.67 − 0.99]
0.87 [0.60 − 0.98]
0.92 [0.64 − 1.00]
1.00 [0.59 − 1.00]
0.62 [0.38 − 0.82]
1.00 [0.84 − 1.00]
0.96 [0.79 − 1.00]
1.00 [0.72 − 1.00]
1.00 [0.75 − 1.00]
0.88 [0.62 − 0.98]
0.92 [0.62 − 1.00]
0.92[0.89 − 0.95]
0.2
1.0
SENSITIVITY

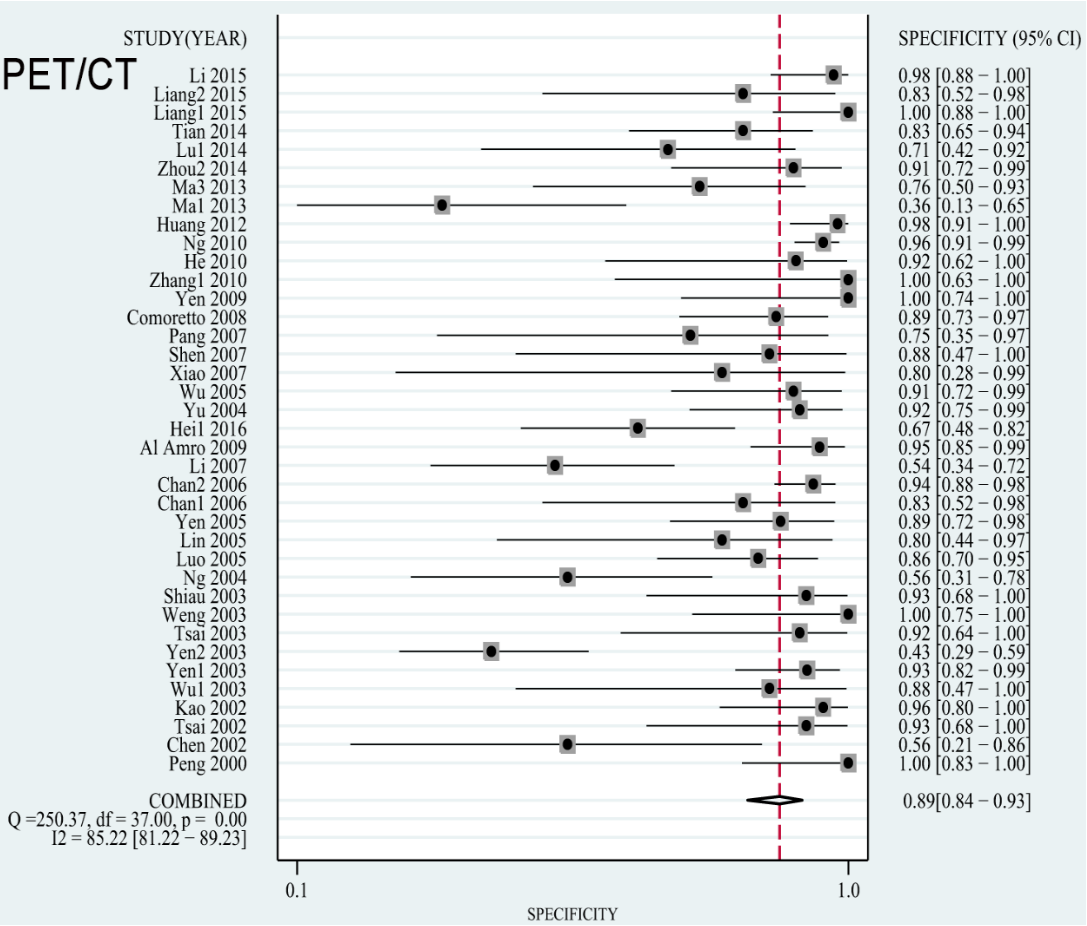
STUDY(YEAR)
PET/CT
Li 2015
Liang2 2015
Liang1 2015
Tian 2014
Lu1 2014
Zhou2 2014
Ma3 2013
Ma1 2013
Huang 2012
Ng 2010
He 2010
Zhang1 2010
Yen 2009
Comoretto 2008
Pang 2007
Shen 2007
Xiao 2007
Wu 2005
Yu 2004
Hei1 2016
Al Amro 2009
Li 2007
Chan2 2006
Chan1 2006
Yen 2005
Lin 2005
Luo 2005
Ng 2004
Shiau 2003
Weng 2003
Tsai 2003
Yen2 2003
Yen1 2003
Wu1 2003
Kao 2002
Tsai 2002
Chen 2002
Peng 2000
COMBINED
Q =250.37, df = 37.00, p = 0.00
I2 = 85.22 [81.22 − 89.23]
SPECIFICITY (95% CI)
0.98 [0.88 − 1.00]
0.83 [0.52 − 0.98]
1.00 [0.88 − 1.00]
0.83 [0.65 − 0.94]
0.71 [0.42 − 0.92]
0.91 [0.72 − 0.99]
0.76 [0.50 − 0.93]
0.36 [0.13 − 0.65]
0.98 [0.91 − 1.00]
0.96 [0.91 − 0.99]
0.92 [0.62 − 1.00]
1.00 [0.63 − 1.00]
1.00 [0.74 − 1.00]
0.89 [0.73 − 0.97]
0.75 [0.35 − 0.97]
0.88 [0.47 − 1.00]
0.80 [0.28 − 0.99]
0.91 [0.72 − 0.99]
0.92 [0.75 − 0.99]
0.67 [0.48 − 0.82]
0.95 [0.85 − 0.99]
0.54 [0.34 − 0.72]
0.94 [0.88 − 0.98]
0.83 [0.52 − 0.98]
0.89 [0.72 − 0.98]
0.80 [0.44 − 0.97]
0.86 [0.70 − 0.95]
0.56 [0.31 − 0.78]
0.93 [0.68 − 1.00]
1.00 [0.75 − 1.00]
0.92 [0.64 − 1.00]
0.43 [0.29 − 0.59]
0.93 [0.82 − 0.99]
0.88 [0.47 − 1.00]
0.96 [0.80 − 1.00]
0.93 [0.68 − 1.00]
0.56 [0.21 − 0.86]
1.00 [0.83 − 1.00]
0.89[0.84 − 0.93]
0.1
1.0
SPECIFICITY

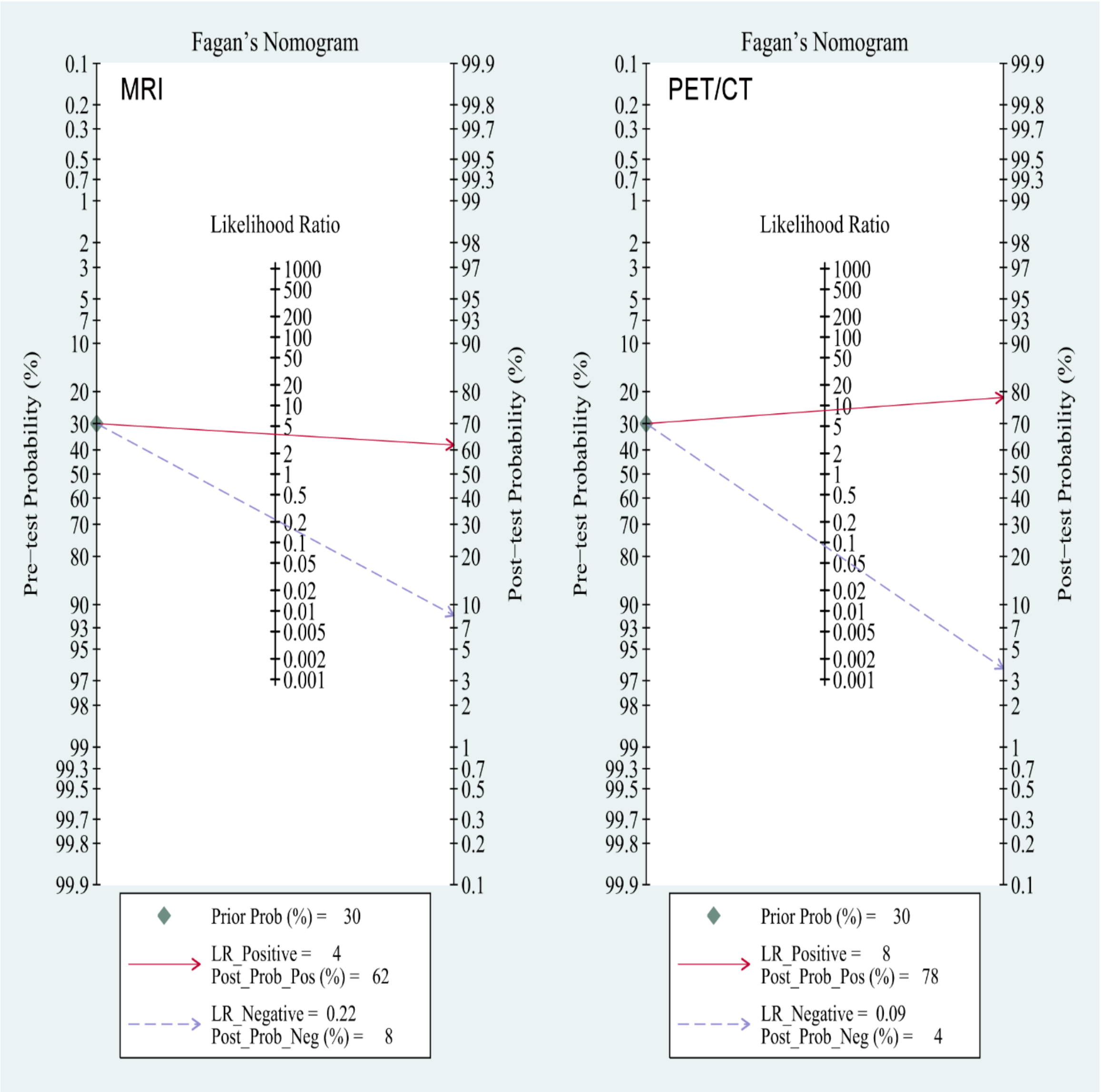
Fagan's Nomogram
MRI
Likelihood Ratio
Pre−test Probability (%)
Post−test Probability (%)
Prior Prob (%) = 30
LR_Positive = 4
Post_Prob_Pos (%) = 62
LR_Negative = 0.22
Post_Prob_Neg (%) = 8
Fagan's Nomogram
PET/CT
Likelihood Ratio
Pre−test Probability (%)
Post−test Probability (%)
Prior Prob (%) = 30
LR_Positive = 8
Post_Prob_Pos (%) = 78
LR_Negative = 0.09
Post_Prob_Neg (%) = 4
